# Sex Differences in Cortical Structural Alterations in Major Depressive Disorder With Suicidal Ideation

**DOI:** 10.1155/da/1706750

**Published:** 2025-07-02

**Authors:** Jiayi Liu, Hongyong Zhang, Yang Wu, Wu Li, Meng Li, Xiaochi Yuan, Cong Zhou, Min Chen, Jingjing Zhou, Jian Cui

**Affiliations:** ^1^Department of Psychiatry, Jining Medical University, Jining 272000, China; ^2^Department of Psychiatry, Shandong Daizhuang Hospital, Jining 272000, China; ^3^Medical Imaging Department, Shandong Daizhuang Hospital, Jining 272000, China; ^4^Department of Equipment, Shandong Daizhuang Hospital, Jining 272000, China; ^5^The National Clinical Research Center for Mental Disorders and Beijing Key Laboratory of Mental Disorders, Beijing Anding Hospital and the Advanced Innovation Center for Human Brain Protection, Capital Medical University, Beijing 100088, China; ^6^Precision Medicine Laboratory, Shandong Daizhuang Hospital, Jining 272000, China

**Keywords:** cortical structure, major depressive disorder, SBM, suicidal ideation

## Abstract

**Background:** Previous studies have documented brain structural changes in major depressive disorder with suicidal ideation (MDDSI). While sex differences in brain structure have been observed in MDD, the specific differences in MDDSI remain underexplored. This study aims to examine sex-specific cortical structural changes in MDDSI patients, with the goal of improving suicide risk assessment and supporting the development of sex-specific interventions.

**Methods:** High-resolution T1-weighted magnetic resonance imaging (MRI) data were acquired from 177 participants, including 117 patients with MDD (44 males and 73 females) and 60 healthy controls (HC; 23 males and 37 females). Using FreeSurfer, we assessed the main effects of both group and sex, as well as sex-by-group interactions, on cortical thickness (CT), surface area (SA), cortical volume (CV), and local gyrification index (LGI). Pearson partial correlation analysis was performed to examine the associations between sex-specific regions and SI scores.

**Results:** Patients with MDDSI exhibited significantly reduced LGI in the right inferior and superior parietal cortices, irrespective of sex. Significant sex-by-group interactions were identified and post hoc analyses revealed that male MDDSI patients showed significantly greater CT in the right lingual gyrus (LG) compared to their female counterparts, along with smaller SA in the right lateral occipital cortex (LOC) and reduced CV in the right pericalcarine cortex (PCAL) compared to their sex-matched MDD patients without SI (MDDNonSI). No significant associations were found between these structural changes and SI scores.

**Conclusion:** This study highlights sex-specific differences in cortical structures in MDDSI patients. These findings contribute to understanding the neurobiological mechanisms underlying MDD with SI.

**Trial Registration:** Chinese Clinical Trial Registry (ChiCTR): ChiCTR2100049646

## 1. Introduction

Major depressive disorder (MDD) is a significant global health concern, affecting over 280 million individuals worldwide and contributing substantially to disability and mortality rates [1, 2]. Among individuals diagnosed with MDD, the risk of suicidality is notably elevated, which include suicidal ideation (SI), suicide plan, suicide attempt, and completed suicide [3]. SI, defined as thoughts or plans related to suicide, is a critical predictor of future suicidal actions and poses a serious threat to an individual's social functioning [4, 5]. Alarmingly, nearly all individuals who attempt suicide report experiencing prior suicidal thoughts [6]. Furthermore, suicide plays an important role in the development of treatment-resistant depression in patients with MDD, which subsequently extends the time required for sustained recovery [7]. These facts highlight the urgency of understanding the underlying mechanisms of MDD and SI to develop effective prevention strategies.

Sex differences in incidence rates and clinical characteristics have been well-documented in MDD and SI. Females are more likely to experience depression at significantly higher rates [8, 9]. They also typically present with more severe depressive symptoms [10], which might be potentially influenced by hormonal fluctuations and social roles [11, 12]. In contrast, males show more severe SI, have higher rates of completed suicides, and are more inclined to use more lethal and violent methods [13, 14].

Several neuroimaging studies have highlighted sex differences in cortical structural changes in individuals with MDD. In terms of gray matter volume (GMV), male patients exhibited significant increases in the left cerebellum, alongside reductions in the bilateral middle temporal gyrus and the left ventral medial prefrontal gyrus. In contrast, female patients showed GMV reductions specifically in the left lingual gyrus (LG) and the dorsal medial prefrontal gyrus [15]. Regarding surface area (SA), female patients had significantly smaller SA in regions such as the right superior frontal gyrus, medial orbitofrontal gyrus, inferior frontal gyrus triangle, superior temporal gyrus, middle temporal gyrus, lateral occipital gyrus, and inferior parietal lobule compared to male patients [16]. In the cortical volume (CV) of right rostromedial prefrontal cortex and local gyrification index (LGI) of left visual cortex, female patients showed lower SA and LGI compared with sex-matched healthy controls (HC), whereas the opposite patterns of significant effects were seen in male [17]. These findings collectively emphasize the distinct patterns of brain structural changes between sex in MDD. Furthermore, structural brain changes have been also found in MDDSI patients, particularly in the fronto-limbic circuit, which includes key regions such as the ventromedial prefrontal cortex, ventrolateral prefrontal cortex, dorsomedial prefrontal cortex, orbitofrontal cortex, amygdala, hippocampus, and anterior cingulate cortex [18–24]. These regions are essential for emotional regulation, decision-making, reward processing, and memory formation, all of which contribute to shaping behavior and emotional responses [25–27]. However, to the best of our knowledge, research that comprehensively examines sex differences in brain morphometric features among individuals with MDDSI remains limited, underscoring the need to understand sex-specific patterns in these conditions.

Surface-based morphometrics (SBM) [28, 29] and voxel-based morphometrics (VBM) [30] are widely used techniques for evaluating cortical morphological measures and have been extensively applied in neuroimaging studies examining brain structural changes in depression. While VBM analyzes GMV, it is limited by voxel size, which reduces its sensitivity to detailed cortical features such as cortical thickness (CT), SA, and LGI [31]. SBM techniques address these limitations by capturing complex cortical features with greater precision. FreeSurfer [32], a commonly used tool for SBM analysis, offers higher accuracy in cortical segmentation and fine structural analysis, making it the preferred tool for this study.

Based on the above, this study aims to expand on existing research by using SBM to investigate sex differences in cortical morphometric measures, focusing specifically on brain regions implicated in MDD and SI. In addition, the study will explore how these sex-specific structural differences correlate with the severity of SI. Through these analyses, we hope to offer novel neurobiological insights that can enhance suicide risk assessment in MDD and inform the development of more tailored, sex-sensitive intervention strategies.

## 2. Materials and Methods

### 2.1. Participants

A total of 180 participants were enrolled in this study conducted at Shandong Daizhuang Hospital from April 2022 to December 2023, including 120 MDD patients (45 males and 75 females) and 60 HC (23 males and 37 females). Participants were categorized according to their biological sex assigned at birth (male or female). The inclusion criteria for the MDD group, adapted from our previous study [33] with minor modifications, were as follows: (1) aged 18–65, regardless of sex; (2) MDD diagnosis confirmed by Mini International Neuropsychiatric Interview (MINI) based on DSM-V criteria, including both first-episode and recurrent MDD without psychotic symptoms; (3) a total score of ≥14 on the 17-item Hamilton Depression Rating Scale (HAMD-17) at screening; (4) a minimum of primary school education with sufficient cognitive ability to comprehend the assessment scales employed in the study; (5) native Han Chinese and right-handed as determined by the Edinburgh Handedness Inventory, to minimize potential confounding effects of ethnicity on brain structure, given prior studies indicating significant cortical differences across ethnic groups [34, 35]; (6) no previous systematic use of antidepressants; if used, for no more than 7 days in the past 14 days; (7) no history of other psychiatric comorbidities; (8) not pregnant or lactating; (9) no significant physical or neurological conditions, including epilepsy or head trauma; (10) no contraindications for magnetic resonance imaging (MRI) scanning. The HC group was matched with the MDD group for age and sex and confirmed to have no DSM-V psychiatric diagnosis via MINI assessment. All participants included in the study underwent MRI scans, and three patients from the MDD group were excluded due to excessive head motion during the scans. Ultimately, 177 participants were included in the final analysis. The study was approved by the Ethics Committee of Daizhuang Hospital, Shandong, China, and written informed consent was obtained from all participants.

### 2.2. Assessment of Depression and Suicidal Ideation

The Hamilton Depression Rating Scale (HAMD-17) [36] was utilized to assess the severity of depression. The 14-item Hamilton Anxiety Rating Scale (HAMA-14) was used to evaluate anxiety severity. The Beck Scale for Suicidal Ideation (BSI), developed by Beck et al. [37] in 1979, was used to assess the severity of SI in patients. SI was also assessed using HAMD item-3 [38, 39]. In the present study, the presence of SI was defined by a score of two or higher on HAMD item-3, following criteria widely used in previous research [40–42]. Based on this definition, MDD patients were subsequently divided into two groups: the MDD with SI group (MDDSI, *n* = 57; 14 males, 43 females) and the MDD without SI group (MDDNonSI, *n* = 60; 30 males, 30 females).

### 2.3. MRI Data Acquisition

High-resolution structural images were acquired using a Siemens 3.0 T MAGNETOM Lumina MRI scanner (Siemens Medical Solutions, Erlangen, Germany) equipped with a 32-channel head coil, following a magnetization-prepared rapid gradient-echo (MPRAGE) T1-weighted sequence. The main parameters were as follows: repetition time (TR) = 2530 ms, echo time (TE) = 1.85 ms, flip angle = 15°, field of view (FOV) = 256 × 256 mm^2^, slice number = 192, and voxel size = 1 × 1 × 1 mm^3^. These parameters were based on those from our prior study [33], ensuring methodological continuity.

### 2.4. Quality Control of MRI

The procedures followed for MRI image quality control were consistent with those described in our previous research [33]. Before the MRI scan, participants were informed about the scanning process. During the scan, they were instructed to relax, keep their eyes closed, remain awake, and minimize head movement. Foam cushioning was placed around the head to limit motion, and soft earplugs were provided to reduce scanner noise. Participants were given the option to stop the procedure if they felt uncomfortable. Immediately after image acquisition, radiologists visually inspected the data. Three MDD patients were excluded from the analysis due to excessive head motion.

### 2.5. Image Processing

Utilizing FreeSurfer software (version 6.0) (http://surfer.nmr.mgh.harvard.edu/) to process T1-weighted images. Firstly, the image is preprocessed through the “recon-all” pipeline in FreeSurfer. The standard pipeline included motion correction and averaging, removal of non-brain tissue, automated Talairach transformation, segmentation of subcortical structures, intensity normalization, tessellation of the gray/white matter boundary, automated topology correction, surface deformation, and inflation, registration to a spherical atlas, cerebral cortex parcellation, creation of surface-based data, and CT calculation [43–48]. Subsequently, quality checks were conducted on the processing results, including Talairach registration, skull removal, and white matter segmentation. Fifteen participants with image registration biases, including six cases of MDDSI, two cases of MDDNonSI, and seven cases of HC, were fixed manually and re-run to ensure the accuracy of the data. Finally, during the data processing, we also extracted the values of estimated total intracranial volume (eTIV) for each participant, which were utilized for subsequent statistical analyses.

### 2.6. Statistical Analysis

Statistical analysis of cortical morphometrics was performed with the “command-line” group analysis stream in FreeSurfer (https://surfer.nmr.mgh.harvard.edu/fswiki/FsTutorial/GroupAnalysis). First, for the analysis of CT, SA, and CV, we applied a 10 mm smoothing kernel. The LGI was not smoothed because smoothing might obscure local gyrification features [17, 49]. Second, the main effects and sex-by-group interaction effects on CT, SA, CV, and LGI were analyzed using a general linear model (GLM) in a vertex-by-vertex manner, with group and sex as fixed factors, controlling for age, years of education, eTIV and HAMD total scores. A cluster-wise correction for multiple comparisons was performed with precomputed Z Monte Carlo simulation [50], setting the significance level at cluster-wise *p*-values (CWP) less than 0.05 [51]. Significant clusters showing the main effects of group and sex-by-group interactions were defined as regions of interest (ROIs). The mean values from these ROIs were extracted for post hoc analyses and partial correlations with clinical characteristics.

Statistical analyses of clinical and demographic characteristics were conducted using the Statistical Package for the Social Sciences (SPSS 22.0 for Windows). The chi-square test was conducted for the categorical variable of sex. One-way analysis of variance (ANOVA) was utilized for continuous variables, including age, years of education, eTIV, HAMD scores, and HAMA scores to examine whether there were differences between groups. An independent-samples *t*-test was conducted to compare the illness duration between relevant groups. Post hoc pairwise comparisons were conducted using the least significant difference (LSD) *t*-test (equal variances assumed) or Dunnett's T3 test (equal variances not assumed). The significance level was set at *α* = 0.05 for a two-tailed test and *p* < 0.05 was taken to indicate statistical significance.

Furthermore, Pearson partial correlation analysis was performed to examine the correlations between ROIs and HAMD item-3 scores, controlling for age, years of education, eTIV and HAMD-17 scores minus HAMD item-3 scores [49]. The false discovery rate (FDR) correction was applied, and statistical significance was set at *p* < 0.05.

## 3. Results

### 3.1. Demographic and Clinical Characteristics

Sex-specific demographic and clinical features of the 57 MDD patients with SI (MDDSI, 14 males and 43 females), 60 MDD patients without SI (MDDNonSI, 30 males and 30 females) and 60 HC (23 males and 37 females) are shown in Table 1. The results indicated that without considering sex, there were no significant differences in age or eTIV among the three groups (all *p*-values > 0.05). Additionally, no significant difference in the duration of illness was found between the MDDSI and MDDNonSI groups (independent-samples *t*-test, *p* > 0.05). However, significant differences were found among the three groups in terms of sex composition, education years, HAMA scores, and HAMD scores. Post hoc analysis showed that the MDDSI group had significantly higher HAMD scores compared to the MDDNonSI group (Dunnett's T3 test, *p* < 0.05). To account for potential confounding, HAMD scores were included as covariates in further analyses.

### 3.2. Significant Main Effects of Group of Cortical Structural Alterations Between MDDSI and MDDNonSI Group

**Figure 1:**
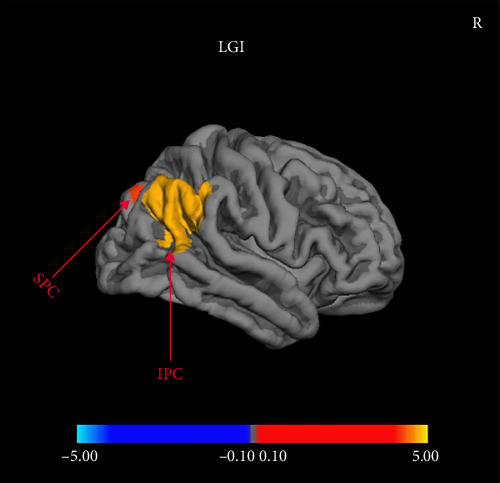
Regions with significant main effects of group between NonSI and SI on local gyrification index of right inferior and superior parietal cortex. IPC, inferior parietal cortex; LGI, local gyrification index; R, right; SPC, superior parietal cortex. The color-bar for *p* values was on a logarithmic scale (log10) with a range of 0.10–5.00.

Compared with MDDNonSI, MDDSI patients showed significantly reduced LGI in right inferior and superior parietal cortex regardless of sex (Figure 1). There was no significant difference in CT, SA, and CV between MDDSI and MDDNonSI group (FDR set at *p* < 0.05).

### 3.3. Significant Sex-by-Group Interactions of Cortical Structural Alterations in MDDSI and MDDNonSI Group

**Figure 2:**
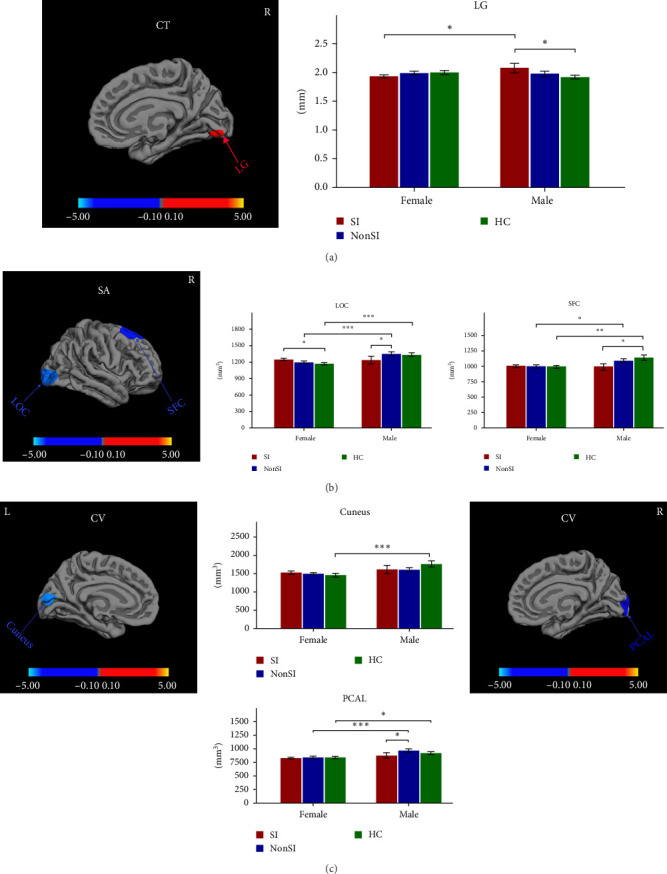
Regions with significant sex-by-group interactions in cortical structures in SI and NonSI. Significant sex-by-group interactions on CT of right LG (A), SA of right LOC and right SFC (B), CV of left cuneus and right PCAL (C) between SI and NonSI (cluster-wise probability <0.05). The color-bar for *p* values was on a logarithmic scale (log10) with a range of 0.10–5.00. These effects are illustrated by bar charts. For the post hoc analyses, *⁣*^*∗*^ indicates *p* < 0.05; *⁣*^*∗∗*^ indicates *p* < 0.01; *⁣*^*∗∗∗*^ indicates *p* < 0.001. HC, healthy controls; *L*, left; LG, lingual gyrus; LOC, lateral occipital cortex; SI, MDD with suicidal ideation; NonSI, MDD without suicidal ideation; PCAL, pericalcarine cortex; *R*, right; SFC, superior frontal cortex. (A) Sex-by-group interactions on cortical thickness of right lingual gyrus. (B) Sex-by-group interactions on cortical surface area of right lateral occipital cortex and right superior frontal cortex. (C) Sex-by-group interactions on cortical volume of left cuneus and right pericalcarine cortex.

Significant sex-by-group interactions were observed in CT of right LG, SA of right lateral occipital cortex (LOC) and right superior frontal cortex (SFC), and CV of left cuneus and right pericalcarine cortex (PCAL) (Table 2, Figure 2). No significant differences were observed in the LGI. In the CT of right LG, subsequent post hoc analyses revealed that male MDDSI group showed significantly increased CT relative to both the female MDDSI and male HC groups, whereas no significant differences were found between MDDSI and MDDNonSI groups within the same sex (Figure 2A, post hoc *p* < 0.05). Regarding the SA of the right LOC and right SFC, distinct sex-specific patterns emerged. The male MDDSI group showed significantly reduced SA in the right LOC compared to the sex-matched MDDNonSI group, while no significant differences were observed in females. In contrast, the female MDDSI group exhibited a significantly larger SA in the right LOC compared to female HC group. Although no similar patterns were found in the SFC region, the male MDDSI group demonstrated reduced SA relative to male HC group (Figure 2B, post hoc *p* < 0.05). Finally, post hoc analyses of CV in the left cuneus and right PCAL showed no significant differences in the left cuneus between MDDSI subgroups of different sex, nor between MDDSI and MDDNonSI groups of the same sex. However, the male MDDSI group exhibited significantly reduced CV in the right PCAL compared to the sex-matched MDDNonSI group (Figure 2C, post hoc *p* < 0.05).

### 3.4. Partial Correlations With Clinical Features

**Figure 3:**
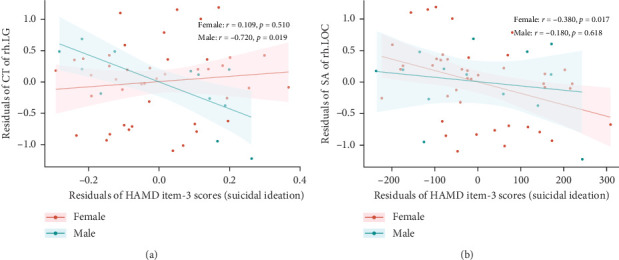
Partial correlation analysis between HAMD item-3 (suicidal ideation) scores and regions with significant sex-by-group interactions in cortical structures. Residual plots showed the correlations of CT in the right LG (A), SA in the right LOC (B) with HAMD item-3 scores in female and male individuals with SI separately, controlling for age, education years, eTIV, and HAMD total scores minus the scores of item-3 (suicidal ideation). The presented *p* values for partial correlation analyses did not pass the FDR correction. CT, cortical thickness; LG, lingual gyrus; LOC, lateral occipital cortex; rh, right hemisphere; SA, surface area. (A) Partial correlation of CT of rh.LG with HAMD item-3 scores. (B) Partial correlation of SA of rh.LOC with HAMD item-3 scores.

After controlling for age, years of education, eTIV, and HAMD-17 total scores minus HAMD item-3 (SI) scores, differences were found in the correlations between suicide scores (HAMD item-3) and specific brain measures between males and females within the MDDSI group. For the CT of the right LG, males showed a significant negative correlation (*r* = −0.720, *p*=0.019), but this was no longer significant after multiple comparisons correction (corrected *p*-value = 0.094). Females did not show a significant correlation (*r* = 0.109, *p*=0.510, corrected *p*-value = 0.541) (Figure 3A). For the SA of right LOC, males showed no significant correlation (*r* = −0.180, *p*=0.618, corrected *p*-value = 0.999), while females displayed a significant negative correlation (*r* = −0.380, *p*=0.017), though this did not survive after correction (corrected *p*-value = 0.085) (Figure 3B).

## 4. Discussion

To our knowledge, this study is the first comprehensive investigation of sex-specific cortical structural alterations in a relatively large cohort of individuals with MDD with SI (MDDSI). We identified both common and sex-specific changes in cortical morphometry among individuals with MDDSI. Three major findings emerged. First, MDDSI patients exhibited a decreased LGI in the right inferior and superior parietal cortices compared to those without SI (MDDNonSI). Second, significant sex-related differences were observed in the SA of the right LOC and SFC, the CV of the right PCAL and left cuneus, and the CT of the right LG. Third, correlations between clinical variables and cortical morphometry differed by sex but did not survive correction for multiple comparisons. These findings provide novel insights into the neuroanatomical alterations associated with SI in depression and highlight the importance of further investigations into sex-specific differences in cortical structure.

In this study, we observed reduced LGI in the right inferior and superior parietal cortices among MDDSI patients compared with MDDNonSI patients, suggesting that cortical folding alterations in these regions may be associated with the presence of SI. The parietal cortex is a critical region for cognitive control, emotional regulation, and spatial perception [52]. As a measure of cortical folding during brain maturation [17, 53], reduced LGI may reflect limitations in cortical development, reduced neural connectivity, or functional impairments [54, 55]. These alterations may weaken the brain's ability to regulate negative emotions or adaptively manage stress, thereby increasing vulnerability to SI. Our findings differ from those of Li et al. [56], who reported reduced parietal sulcal depth but no LGI differences between MDDSI and MDDNonSI patients. This discrepancy might stem from variations in imaging analysis techniques or sample characteristics. Despite inconsistencies across previous studies [17, 57–59], our results reinforce the emerging view that the parietal cortex plays a key role in the neuropathology of SI. These findings suggest that LGI in the parietal cortex may serve as a structural marker for identifying individuals at high suicide risk, laying a foundation for future research on neurobiological targets for prevention and early intervention.

We further examined the sex-by–group interaction effects on specific cortical metrics. Post hoc analyses revealed that male MDDSI patients exhibited significantly reduced SA of the right LOC and CV of the right PCAL compared to sex-matched MDDNonSI patients. They also showed increased CT in the right LG relative to their female counterparts. Notably, these alterations were not seen in females, suggesting a potential male-specific pattern of cortical remodeling associated with suicide vulnerability. The LOC, PCAL, and LG are key components of the visual cortex, involved not only in visual processing but also in emotional regulation [60–62]. Our findings of SA, CV, and CT alterations in these regions could be related to impairments in processing visual information previously reported in MDD and SI [63, 64]. These abnormalities may also contribute to altered facial emotion recognition in MDDSI [65]. Accumulating evidence suggests that sex differences in visual processing emerge early in development, with females more vulnerable to pre-attentive visual processing deficits in MDD, whereas males tend to exhibit enhanced sensitivity to visual change detection [66, 67]. These divergent patterns may reflect fundamentally distinct neurodevelopmental trajectories across sexes that could shape visual system involvement in the pathophysiology of depression and SI.

Beyond visual processing, these regions may also influence emotional regulation via their connectivity with limbic structures. The LG, for instance, has been shown to be functionally connected to the amygdala [62], which in MDD shows an emotion-congruent processing bias: hyperactivation to negative stimuli and hypoactivation to positive stimuli [68]. The increased CT in the right LG may amplify this amygdala-driven bias, heightening emotional reactivity and sensitivity to negative emotions. Such disruptions in emotion processing may undermine social functioning, exacerbate feelings of isolation, and reinforce negative self-perceptions, ultimately increasing vulnerability to suicide. Moreover, sex-specific lateralization of amygdala responses during affective face processing has been reported [69], suggesting that structural alterations in regions like the LG may differentially impact emotional regulation networks across sexes.

It is well-established that both normative and pathological neural process operate through complex and interconnected brain networks [70]. The “Suicide GMV damage network” encompass the dorsal default mode network (DMN), basal ganglia, and anterior salience network [71–73], with DMN dysfunction consistently implicated in depression with SI [33, 74]. Reduced resting-state functional connectivity (RSFC) between the LOC and left PCC, along with increased weighted hubness in the right PCAL, has been reported to be associated with elevated suicide risk in depressed adolescents [75, 76]. In this context, the reduced SA in the LOC may reflect diminished functional coupling with the DMN nodes such as the posterior cingulate cortex (PCC), while the decreased CV in the PCAL may indicate reduced network integration. Emerging evidence suggests sex-specific resting-state network dynamics in MDD, with males exhibiting prolonged dominance of DMN activity coupled with salience network suppression [77]. Such functional imbalances may interact with structural alterations in visual and emotional regions, further contributing to sex-related differences in suicide vulnerability. These structure alterations may disrupt DMN-mediated self-referential thought and emotional regulation, potentially contributing to negative cognitive cycles and increased suicide risk [78]. Taken together, increased CT in the right LG, decreased SA in the right LOC and CV in the right PCAL may serve as potential male-specific neural markers of suicide vulnerability in depression. Future longitudinal and multimodal studies are warranted to clarify the mechanisms underlying these sex-specific pathways related to risk and expression of MDD and SI.

Furthermore, we explored the relationship between these cortical structure measures and the severity of SI. Among male MDDSI patients, a negative correlation was observed between CT of the right LG and SI scores, suggesting that reduced CT in right LG may reflect heightened vulnerability to more severe SI in males. In contrast, female MDDSI patients exhibited a negative correlation between the SA of the right LOC and SI scores, indicating that lower SA in the right LOC may be associated with greater severity of SI in females. Unfortunately, these associations did not survive correction for multiple comparisons. Although not statistically robust, these preliminary findings suggest that structural alterations in the right LG and LOC may influence perceptual and emotional processes related to SI, providing a foundation for future studies exploring structural-functional mechanism in SI.

Our study has several limitations that warrant consideration. Firstly, due to the cross-sectional design, we cannot determine causal relationships between changes in cortical measures, suicidal behavior, and depression. Longitudinal studies are needed to explore the dynamic interactions among these factors over time. Secondly, while previous research has highlighted significant structural and functional differences between first-episode and recurrent MDD, we did not differentiate between these groups. However, we controlled for this variable as a covariate to minimize its potential impact on our findings [79]. Future studies should stratify samples to explore these variations more effectively. Thirdly, the sex imbalance in our sample may limit the understanding of sex-specific morphological changes. Future research should aim to include larger, more balanced samples to better assess these differences. Fourthly, we recruited only adult native Han Chinese participants to minimize potential confounding from ethnic and cultural variability. While this approach enhances internal validity, it may limit the generalizability of our findings. Future studies should aim to replicate these results in more demographically diverse populations. Finally, the exclusive reliance on structural MRI in our study may limit the depth of understanding regarding the neural mechanisms underlying MDDSI. Future research integrating multimodal imaging techniques, such as functional MRI (fMRI) or diffusion tensor imaging (DTI), may provide a more nuanced perspective by correlating structural alterations with functional and connectivity data.

## 5. Conclusion

In this study, we conducted a relatively comprehensive assessment of cortical morphometry in individuals with MDDSI, uncovering both sex-specific and sex-neutral anatomical alterations. The most pronounced sex-related differences were observed in the occipital cortex, particularly in SA, CV, and CT, which may reflect divergent neurodevelopmental trajectories across sexes. These findings highlight the importance of considering sex as a critical biological variable when investigating brain structural changes related to SI. Future research should further explore the neurobiological mechanisms underlying these sex-specific differences, as such insights may contribute to the development of more effective and personalized intervention strategies for treating depression, particularly in individuals with SI.

## Figures and Tables

**Table 1:** Demographic and clinical characteristics for all subjects (*N* = 177).

Variable	SI (*N* = 57)	NonSI (*N* = 60)	HC (*N* = 60)	*p* Values	A (SI vs. NonSI)	B (SI vs. HC)	C (NonSI vs. HC)
Age (years)	26.68 (10.58)	30.37 (9.78)	28.17 (10.03)	0.143	0.051	0.430	0.236
Male	26.64 (10.64)	29.37 (8.12)	23.57 (4.73)	0.011*⁣*^*∗*^	0.780	0.673	0.006*⁣*^*∗∗*^
Female	26.70 (10.69)	31.37 (11.25)	31.03 (11.37)	0.121	0.079	0.084	0.901
Sex (male/female)	14/43	30/30	23/37	0.018*⁣*^*∗*^	0.007*⁣*^*∗*^*⁣*^*∗*^	0.177	0.270
Education (years)	12.46 (3.03)	11.43 (3.67)	16.42 (2.10)	<0.001*⁣*^*∗∗∗*^	0.275	<0.001*⁣*^*∗∗∗*^	<0.001*⁣*^*∗∗∗*^
Male	12.57 (2.24)	11.03 (3.52)	16.57 (2.29)	<0.001*⁣*^*∗∗∗*^	0.238	<0.001*⁣*^*∗∗∗*^	<0.001*⁣*^*∗∗∗*^
Female	12.42 (3.27)	11.83 (3.82)	16.32 (2.00)	<0.001*⁣*^*∗∗∗*^	0.871	<0.001*⁣*^*∗∗∗*^	<0.001*⁣*^*∗∗∗*^
eTIV (cm3)	1,473,712.72 (150,460.77)	1,533,096.18 (146,375.28)	1,540,288.77 (183,004.12)	0.053	0.048*⁣*^*∗*^	0.027*⁣*^*∗*^	0.807
Male	1,617,506.65 (125,731.44)	1,614,525.17 (120,605.68)	1,714,124.52 (122,612.58)	0.010*⁣*^*∗*^	0.940	0.023*⁣*^*∗*^	0.005*⁣*^*∗∗*^
Female	1,426,896.09 (127,103.52)	1,451,667.20 (123,797.76)	1,432,228.72 (119,966.82)	0.690	0.402	0.848	0.524
HAMD scores	23.21(4.29)	21.13 (4.56)	0.42 (0.74)	<0.001*⁣*^*∗∗∗*^	0.037*⁣*^*∗*^	<0.001*⁣*^*∗∗∗*^	<0.001*⁣*^*∗∗∗*^
Male	23.79 (5.34)	20.07 (4.39)	0.17 (0.39)	<0.001*⁣*^*∗∗∗*^	0.094	<0.001*⁣*^*∗∗∗*^	<0.001*⁣*^*∗∗∗*^
Female	23.02 (3.95)	22.20 (4.56)	0.57 (0.87)	<0.001*⁣*^*∗∗∗*^	0.808	<0.001*⁣*^*∗∗∗*^	<0.001*⁣*^*∗∗∗*^
HAMA scores	18.21(5.50)	17.13 (4.80)	0.30 (0.79)	<0.001*⁣*^*∗∗∗*^	0.596	<0.001*⁣*^*∗∗∗*^	<0.001*⁣*^*∗∗∗*^
Male	16.86 (5.63)	16.67 (5.29)	0.26 (0.54)	<0.001*⁣*^*∗∗∗*^	0.999	<0.001*⁣*^*∗∗∗*^	<0.001*⁣*^*∗∗∗*^
Female	18.65 (5.45)	17.60 (4.30)	0.32 (0.92)	<0.001*⁣*^*∗∗∗*^	0.736	<0.001*⁣*^*∗∗∗*^	<0.001*⁣*^*∗∗∗*^
Illness duration (months)	28.95 (34.30)	27.67 (33.03)	NA	NA	0.837	NA	NA
Male	17.64 (23.15)	31.10 (37.34)	NA	NA	0.223	NA	NA
Female	32.63 (36.69)	24.23 (28.30)	NA	NA	0.296	NA	NA

*Note:* Data were presented as means (standard deviation), except for sex (male/female) was presented as the number of people. Categorical variables were analyzed using the Chi-square test, continuous variables were analyzed using one-way analysis of variance (ANOVA). *⁣*^*∗∗∗*^ indicates *p* < 0.001; *⁣*^*∗∗*^ indicates *p* < 0.01; *⁣*^*∗*^ indicates *p* < 0.05. A: *T*-test and effect size between depressive patients with suicidal ideation and depressive patients without suicidal ideation; B: *T*-test and effect size between depressive patients with suicidal ideation and healthy controls; C: *T*-test and effect size between depressive patients without suicidal ideation and healthy controls. NonSI, MDD without suicidal ideation; SI, MDD with suicidal ideation.

Abbreviations: eTIV, estimated total intracranial volume; HAMA, Hamilton Anxiety Rating Scale; HAMD, Hamilton Depression Rating Scale; HC, healthy controls; MDD, major depressive disorder; NA, not available.

**Table 2:** Clusters of significant main effects of group and sex-by-group interactions in MDD with and without suicidal ideation.

Cortical morphometric	Cluster location	Direction	MNI coordinates (peak vertex)			Size (mm^2^)	CWP
			*x*	*y*	*z*		
Main effects of group (controlling for age, years of education, eTIV, HAMD total scores, and sex)							
LGI	R inferior parietal cortex	SI < NonSI	43.2	−64.0	33.0	3135.19	<0.001*⁣*^*∗∗∗*^
LGI	R superior parietal cortex	SI < NonSI	25.4	−73.7	29.9	367.77	0.004*⁣*^*∗∗*^
Sex-by-group interactions (controlling for age, years of education, eTIV, and HAMD total scores)							
CT	R lingual gyrus	—	10.0	−85.0	−10.9	421.96	0.018*⁣*^*∗*^
SA	R lateral occipital cortex	—	30.6	−90.8	−5.6	1294.35	<0.001*⁣*^*∗∗∗*^
SA	R superior frontal cortex	—	9.2	31.4	54.1	888.90	0.011*⁣*^*∗*^
CV	L cuneus	—	−5.3	−90.7	7.9	746.36	<0.001*⁣*^*∗∗∗*^
CV	R pericalcarine cortex	—	18.1	−94.6	−1.8	466.10	0.019*⁣*^*∗*^

*Note:* The cluster-based *p*-value corresponds to the peak vertex that showed the greatest statistical difference within a cluster (multiple comparison correction based on Monte Carlo simulation, CWP < 0.05). Size (mm^2^) represents the area of the cluster exhibiting significant effects, measured in square millimeters (mm^2^). *⁣*^*∗∗∗*^ indicates *p* < 0.001; *⁣*^*∗∗*^ indicates *p* < 0.01; *⁣*^*∗*^ indicates *p* < 0.05. L (R), left (right) hemisphere; NonSI, MDD without suicidal ideation; SI, MDD with suicidal ideation.

Abbreviations: CT, cortical thickness; CV, cortical volume; eTIV, estimated total intracranial volume; HAMD, Hamilton Depression Rating Scale; LGI, local gyrification index; SA, surface area.

## Data Availability

The data that support the findings of this study are available from the corresponding author upon reasonable request.

## Supporting Information

Supporting Information This study primarily focuses on sex-specific brain structural changes associated with suicidal ideation. Detailed information on other significant findings, including main effects of group and sex as well as their interactions, is summarized in Table S1. Furthermore, to ensure appropriate care for this high-risk population, all participants underwent standardized clinical evaluation and treatment immediately following MRI acquisition, in accordance with current clinical guidelines. Detailed information on the pharmacological treatments and adjunctive interventions is provided in Table S2. Clinical follow-up outcomes, including treatment response rates and changes in HAMD scores at the 2-week time point, are summarized in Table S3.
