# Supplementary material for: Sex Differences in Cortical Structural Alterations in Major Depressive Disorder With Suicidal Ideation
Source: Depress Anxiety. 2025 Jul 2;2025:1706750. doi: 10.1155/da/1706750 (PMC12240658; doi:10.1155/da/1706750)
Supplement: Supporting Information — This study primarily focuses on sex-specific brain structural changes associated with suicidal ideation. Detailed information on other significant findings, including main effects of group and sex as well as their interactions, is summarized in Table S1. Furthermore, to ensure appropriate care for this high-risk population, all participants underwent standardized clinical evaluation and treatment immediately following MRI acquisition, in accordance with current clinical guidelines. Detailed information on the pharmacological treatments and adjunctive interventions is provided in Table S2. Clinical follow-up outcomes, including treatment response rates and changes in HAMD scores at the 2-week time point, are summarized in Table S3. [file 1706750.f1.docx]

**Supplementary Table S1.** Clusters of significant main effects of group and sex and sex-by-group interactions in MDD with and without suicidal ideation and HC.

| **Cortical morpho-metric** | **Cluster location** | **Direction** | **MNI coordinates (peak vertex)** | | | **Size (mm2)** | **CWP** |
| --- | --- | --- | --- | --- | --- | --- | --- |
|  | | | **x** | **y** | **z** |  | |
| Main effects of group | | | | | | | |
| LGI | L posterior cingulate cortex | SI > HC | -4.3 | -8.5 | 37.8 | 2459.69 | <0.001 |
|  | L medial orbitofrontal cortex | SI > HC | -12.9 | 41.9 | -5.4 | 2220.94 | <0.001 |
|  | R posterior cingulate cortex | SI > HC | 3.7 | -10.7 | 34.0 | 893.71 | <0.001 |
|  | L superior frontal cortex | NonSI > HC | -10.2 | 16.7 | 43.8 | 768.30 | <0.001 |
|  | L superior frontal cortex | NonSI > HC | -5.6 | -11.5 | 53.1 | 581.98 | <0.001 |
|  | L medial orbitofrontal cortex | NonSI > HC | -13.3 | 42.6 | -4.5 | 522.01 | <0.001 |
|  | R superior frontal cortex | NonSI > HC | 12.8 | 0.3 | 42.4 | 1249.52 | <0.001 |
| Main effects of sex | | | | | | | |
| CT | R fusiform gyrus | Male > Female | 31.3 | -57.5 | -10.5 | 609.97 | 0.001 |
|  | R superior temporal gyrus | Male > Female | 46.2 | -12.6 | -10.9 | 579.52 | 0.002 |
| SA | L supramarginal gyrus | Male > Female | -55.8 | -47.1 | 36.4 | 1103.82 | 0.003 |
|  | R pars opercularis | Male > Female | 45.9 | 15.4 | 20.6 | 1375.65 | <0.001 |
|  | R superior parietal cortex | Male > Female | 32.7 | -44.3 | 45.4 | 1243.68 | 0.001 |
|  | R middle temporal gyrus | Male > Female | 64.8 | -34.5 | -8.9 | 1162.12 | 0.002 |
| CV | L supramarginal gyrus | Male > Female | -52.3 | -48.2 | 35.6 | 784.63 | <0.001 |
|  | R supramarginal gyrus | Male > Female | 53.5 | -38.4 | 25.1 | 640.20 | 0.002 |
|  | R pars opercularis | Male > Female | 54.6 | 22.3 | 13.7 | 1383.18 | <0.001 |
|  | R superior parietal cortex | Male > Female | 27.9 | -51.8 | 46.5 | 405.55 | 0.040 |
|  | R rostral middle frontal cortex | Male > Female | 18.8 | 60.0 | 11.7 | 584.09 | 0.005 |
| LGI | L insula | Male > Female | -32.3 | -28.8 | 9.6 | 868.71 | <0.001 |
|  | L caudal middle frontal cortex | Male > Female | -36.0 | 24.6 | 38.3 | 646.51 | <0.001 |
|  | L inferior parietal cortex | Male > Female | -31.0 | -54.3 | 35.1 | 485.91 | <0.001 |
|  | L lateral orbitofrontal cortex | Male > Female | -29.1 | 22.0 | -2.2 | 481.81 | <0.001 |
|  | L rostral middle frontal cortex | Male > Female | -38.0 | 45.0 | 8.9 | 451.66 | <0.001 |
|  | R inferior parietal cortex | Male > Female | 38.3 | -64.3 | 46.8 | 5753.39 | <0.001 |
|  | R medial orbitofrontal cortex | Male > Female | 11.2 | 41.0 | -19.3 | 2165.37 | <0.001 |
|  | R superior temporal gyrus | Male > Female | 49.6 | -32.0 | 10.2 | 1820.46 | <0.001 |
|  | R caudal middle frontal cortex | Male > Female | 41.1 | 15.8 | 49.0 | 756.12 | <0.001 |
|  | R superior frontal cortex | Male > Female | 20.3 | 35.3 | 35.6 | 356.57 | 0.004 |
|  | R fusiform gyrus | Male > Female | 41.7 | -49.3 | -12.8 | 346.94 | 0.005 |
|  | R superior frontal cortex | Male > Female | 21.0 | 8.5 | 62.1 | 291.30 | 0.019 |
|  | R pars opercularis | Male > Female | 35.8 | 10.4 | 13.0 | 251.10 | 0.041 |
| Sex-by-group interactions | | | | | | | |
| CT | L superior frontal cortex | - | -14.3 | 47.1 | 2.4 | 654.41 | 0.001 |
|  | L caudal anterior cingulate cortex | - | -12.1 | 14.7 | 34.1 | 388.28 | 0.027 |
|  | R superior frontal cortex | - | 9.0 | 50.5 | 27.4 | 460.73 | 0.011 |
|  | R caudal anterior cingulata cortex | - | 5.9 | 11.6 | 35.0 | 429.37 | 0.017 |

**Continued Supplementary Table S1.** Clusters of significant main effects of group and sex and sex-by-group interactions in MDD with and without suicidal ideation and HC.

| **Cortical morpho-metric** | **Cluster location** | **Direction** | **MNI coordinates (peak vertex)** | | | **Size (mm2)** | **CWP** |
| --- | --- | --- | --- | --- | --- | --- | --- |
|  |  |  | **x** | **y** | **z** |  |  |
| Sex-by-group interactions | | | | | | | |
| CT | R superior temporal gyrus | - | 45.6 | -13.9 | -10.4 | 999.29 | <0.001 |
|  | R superior temporal gyrus | - | 44.8 | -16.3 | -8.8 | 579.48 | 0.002 |
|  | R posterior cingulate cortex | - | 4.7 | 0.1 | 39.1 | 461.11 | 0.010 |
|  | R precentral gyrus | - | 38.6 | -17.5 | 61.1 | 414.20 | 0.020 |
| SA | L caudal middle frontal cortex | - | -27.0 | -4.3 | 44.5 | 1135.63 | 0.002 |
|  | R lateral occipital cortex | - | 26.1 | -98.1 | -10.0 | 832.77 | 0.019 |
|  | R superior frontal cortex | - | 10.0 | 31.0 | 53.7 | 1261.79 | 0.001 |
|  | R inferior parietal cortex | - | 48.3 | -56.7 | 12.3 | 1161.81 | 0.002 |
| CV | L pars orbitalis | - | -39.6 | 43.6 | -12.1 | 629.81 | 0.002 |
|  | R superior frontal cortex | - | 16.5 | 31.2 | 51.4 | 2211.82 | <0.001 |
|  | R inferior parietal cortex | - | 44.7 | -51.5 | 15.5 | 743.65 | <0.001 |
|  | R rostral middle frontal cortex | - | 23.3 | 58.8 | 11.8 | 392.96 | 0.047 |
| LGI | L caudal middle frontal cortex | - | -34.1 | 24.1 | 45.7 | 465.89 | <0.001 |
|  | L inferior temporal cortex | - | -47.8 | -41.6 | -20.3 | 391.65 | 0.002 |
|  | L rostral middle frontal cortex | - | -44.8 | 23.7 | 31.8 | 484.04 | <0.001 |
|  | R lateral occipital cortex | - | 33.5 | -78.8 | 8.6 | 967.04 | <0.001 |
|  | R rostral middle frontal cortex | - | 22.6 | 56.4 | 16.9 | 962.80 | <0.001 |
|  | R superior frontal cortex | - | 23.2 | 22.2 | 40.9 | 933.26 | <0.001 |
|  | R caudal middle frontal cortex | - | 40.2 | 5.7 | 50.0 | 596.52 | <0.001 |
|  | R rostral middle frontal cortex | - | 42.8 | 31.5 | 30.0 | 547.25 | <0.001 |
|  | R fusiform gyrus | - | 32.8 | -48.2 | -17.5 | 338.86 | 0.006 |
|  | R lingual gyrus | - | 20.8 | -50.7 | -8.3 | 834.70 | <0.001 |
|  | R superior frontal cortex | - | 21.5 | 37.6 | 32.1 | 430.22 | <0.001 |
|  | R medial orbitofrontal cortex | - | 6.1 | 44.6 | -21.4 | 412.16 | 0.001 |

The cluster-based p-value corresponds to the peak vertex that showed the greatest statistical difference within a cluster (multiple comparison correction based on Monte Carlo simulation, CWP<0.05). Size(mm²) represents the area of the cluster exhibiting significant effects, measured in square millimeters (mm²). Abbreviations: L (R), left (right) hemisphere; LGI, Local Gyrification Index; CT, cortical thickness; SA, surface area; CV, cortical volume; SI, MDD with suicidal ideation; NonSI, MDD without suicidal ideation; HC, healthy controls.

**Clinical Evaluation and Treatment Procedures.**

All participants underwent a standardized psychiatric evaluation immediately following MRI acquisition and were referred to the outpatient psychiatric department of Shandong Daizhuang hospital. Treatment was administered according to national and international clinical guidelines. The specific pharmacological and adjunctive treatments are summarized in Supplementary Table S2. A follow-up clinical evaluation was conducted two weeks after treatment initiation to assess treatment response, which was defined as a ≥20% reduction in HAMD scores from baseline. Responders continued with their initial medication regimens and were monitored longitudinally. Non-responders underwent dose adjustments or medication changes. For patients with inadequate medication response, adjunctive physical therapies such as repetitive transcranial magnetic stimulation (rTMS) were considered based on clinical judgment. Detailed follow-up outcomes, including response rates and HAMD score changes, are presented in Supplementary Table S3.

**Supplementary Table S2.** Pharmacological and Adjunctive Treatments.

| **Category** | **Medication Examples** | **Indications** |
| --- | --- | --- |
| Antidepressants | Escitalopram, Sertraline, Venlafaxine | Depressive symptoms |
| Hypnotics | Lorazepam, Diazepam | Sleep disturbances |
| Anxiolytics | Tandospirone | Significant anxiety |
| Antipsychotics | Quetiapine, Olanzapine | Psychotic features |
| Adjunctive Physical Therapy | rTMS | Inadequate response to medication |

Abbreviations: rTMS, repetitive transcranial magnetic stimulation.

**Supplementary Table S3.** Clinical Characteristics and Treatment Response in Two-Week Follow-Up in MDD Patients With and Without Suicidal Ideation.

| **Measure** | **MDDSI** | **MDDNonSI** | **Total** |
| --- | --- | --- | --- |
| Total Patients at Baseline | 57 | 60 | 117 |
| Lost to follow-up count | 4 | 2 | 6 |
| Completed Evaluation | 53 | 58 | 111 |
| Baseline HAMD scores | 23.23(4.36) | 21.05(4.54) | - |
| HAMD scores at Week 2 | 13.17(5.62) | 11.55(5.02) | - |
| Mean Reduction Rate | 0.42(0.25) | 0.44(0.23) | - |
| Responders | 42 | 50 | 92 |
| Non-Responders | 11 | 8 | 19 |

Data were presented as the number of people, except for Baseline HAMD scores, HAMD scores at Week 2, and Mean Reduction Rate were presented as means (standard deviation). Responders were defined as patients who achieved a ≥20% reduction in HAMD scores from baseline after 2 weeks of treatment.
